# Monoammonium glycyrrhizinate ameliorates mitochondrial dysfunction-mediated oxidative stress and neuroinflammation via the NRF2/NQO1 axis after spinal cord injury

**DOI:** 10.1080/13510002.2025.2585221

**Published:** 2025-11-15

**Authors:** Tianyi Wang, Jiale Huang, Jian Zhou, Mingjie Xia, Zheng Zhou, Qianqiu Li, Guanhua Xu, Zhanyang Qian, Zhiming Cui

**Affiliations:** a Department of Orthopedics, The Second Affiliated Hospital of Nantong University, Nantong First People's Hospital, Nantong, People’s Republic of China; b Research Institute for Spine and Spinal Cord Disease of Nantong University, Department of Orthopedics, The Second Affiliated Hospital of Nantong University, Nantong First People's Hospital, Nantong, People’s Republic of China; c Key Laboratory of Neuroregeneration of Jiangsu and Ministry of Education, Co-innovation Center of Neuroregeneration, Nantong University, Nantong, People’s Republic of China

**Keywords:** Spinal cord injury, monoammonium glycyrrhizinate, mitochondrial dysfunction, neuroinflammation, oxidative stress, reactive oxygen species, NRF2/NQO1 pathway, microglia

## Abstract

**Background:**

Spinal cord injury (SCI)-induced mitochondrial dysfunction in microglia exacerbates neuroinflammation and neurological deficits. Monoammonium glycyrrhizinate (MAG), a bioactive liquorice-derived compound, exhibits anti-inflammatory and antioxidant properties; however, its effects on microglial mitochondria remain unknown.

**Methods:**

Mice received a moderate contusion injury at the T10 spinal segment. Histopathology was assessed using Hematoxylin-Eosin, Nissl staining, and Luxol Fast Blue; locomotor recovery was evaluated via the Basso Mouse Scale, hindlimb flexion scoring, and gait footprint analysis. RNA-Seq and molecular docking identified KEAP1/NRF2 signaling. Verification employed qPCR, Western blot, and immunofluorescence. Mitochondrial function was gauged by JC-1 and MitoSOX.

**Results:**

In SCI mice, MAG attenuated neuroinflammation, reduced neuronal tissue loss and demyelination, enhanced neuronal survival, and improved functional recovery. Transcriptomic and molecular docking established that MAG directly activates NRF2, promoting dissociation from KEAP1, nuclear translocation, and induction of NQO1. Pathway enrichment analysis further indicated MAG modulation of mitochondrial regulatory processes. MAG treatment significantly restored mitochondrial function in BV2 cells, improving membrane potential and reducing oxidative stress. Critically, NRF2 inhibition with ML385 abolished MAG's protective effects on anti-inflammatory responses and antioxidant activity.

**Conclusion:**

This study identifies MAG as a novel activator of the KEAP1/NRF2/NQO1 axis, alleviating microglial mitochondrial dysfunction and neuroinflammation post-SCI. These findings provide mechanistic insights into MAG's neuroprotective actions and support its therapeutic potential.

## 1. Introduction

Spinal cord injury (SCI) constitutes a devastating insult to the central nervous system (CNS), frequently resulting in irreversible deficits in both sensory and motor functions[1–3]. The pathology evolves through two distinct phases: an initial primary mechanical injury characterized by vascular disruption, axonal shearing, and loss of neuronal membrane integrity; followed by a secondary injury phase involving complex processes including ischemia-hypoxia, inflammation, oxidative stress, and mitochondrial dysfunction[2–4]. During secondary injury, excessive neuroinflammation coupled with redox imbalance initiates a self-perpetuating cycle that synergistically amplifies neural tissue damage[5–7]. This inflammatory-oxidative cascade critically disrupts mitochondrial function via membrane potential collapse, electron transport chain impairment, and permeability transition pore opening, thereby establishing a fundamental barrier to neurological recovery by compromising cellular bioenergetics and activating degenerative pathways[8, 9].

Microglia, the primary CNS immune sentinels, exhibit dual roles in SCI pathogenesis by driving neuroinflammation and oxidative stress upon activation. Following injury, microglia rapidly activate, triggering a deleterious cascade marked by excessive production of interleukin-1β (IL-1β), tumor necrosis factor-α (TNF-α), interleukin-6 (IL-6) and mitochondrial reactive oxygen species (mtROS) [10, 11]. These mediators establish an autocatalytic feedback loop wherein neuroinflammation potentiates oxidative stress (particularly mtROS), which further amplifies inflammatory responses. This vicious cycle culminates in irreversible damage including mitochondrial failure, neuronal apoptosis, and glial scar formation[12–16]. Critically, activated microglia markedly upregulate two key inflammatory enzymes: inducible nitric oxide synthase (iNOS) and cyclooxygenase-2 (COX-2). iNOS catalyzes excessive nitric oxide (NO) production, which reacts with superoxide to form peroxynitrite (ONOO^–^) – a potent oxidant causing protein nitration and membrane lipid peroxidation. COX-2, meanwhile, drives the conversion of arachidonic acid to pro-inflammatory prostaglandins (notably PGE₂), further amplifying neuroinflammation through EP receptor signaling and exacerbating mitochondrial dysfunction. Thus, iNOS and COX-2 act in concert as critical amplifiers within the neuroinflammation-oxidative stress axis post SCI[17, 18]. The sustained microglial activation and consequent neuroinflammation-mitochondrial oxidative stress cascade constitute a pivotal therapeutic target for SCI intervention, as modulating this pathological axis may mitigate secondary damage and enhance functional recovery.

Monoammonium glycyrrhizinate (MAG), the principal bioactive compound derived from glycyrrhiza uralensis root, exhibits potent anti-inflammatory and antioxidant activities[19–22]. Substantial evidence indicates that glycyrrhizic acid (GA) and its derivatives confer therapeutic benefits across diverse pathologies by targeting core inflammatory and oxidative stress pathways. In acute lung injury, GA attenuates pulmonary inflammation through NF-κB inhibition and modulation of the miR-155/SOCS1 axis[23]. In myocardial ischemia/reperfusion injury, GA mitigates cardiac damage via regulation of the Hippo/YAP pathway[24]. For ischemic stroke, GA exerts neuroprotection by suppressing HMGB1/TLR4/NF-κB signaling while activating the Kelch-like ECH-associated protein 1(KEAP1)/Nuclear factor erythroid 2-related factor 2(NRF2) antioxidant response[25]. In Alzheimer’s disease, GA derivatives reduce cognitive impairment by counteracting oxidative neuronal injury[26]. Collectively, these findings position GA as a putative multi-target therapeutic candidate for neuroinflammatory and neurodegenerative disorders. While preliminary evidence suggests MAG may attenuate post-SCI inflammation, its specific mechanisms governing microglial polarization and mitochondrial dysfunction resolution remain undefined.

In recent years, a growing body of research has highlighted the central role of the NRF2 pathway in regulating neuroinflammation. For instance, in an Alzheimer's disease model, Forsythoside A was shown to significantly suppress ferroptosis-mediated neuroinflammation and alleviate pathological damage by activating the NRF2 axis[27]. Similarly, astrocyte-targeting therapy effectively ameliorated neuroinflammation-associated cognitive impairment by enhancing NRF2 pathway activity[28]. In an intracerebral hemorrhage model, intermittent fasting markedly reduced neuroinflammation levels via the NRF2 pathway[29]. Furthermore, itaconate activated an NRF2-dependent anti-neuroinflammatory and neurogenic mechanism via the gut-brain axis, mitigating postoperative cognitive dysfunction[30]. Collectively, these studies demonstrate that activation of the NRF2 pathway effectively inhibits neuroinflammation, mitigates oxidative stress, and promotes neural repair across various neurological disorders. Consequently, targeting the NRF2 signaling axis has emerged as a crucial therapeutic strategy for neuroinflammation-related diseases.

This study elucidates MAG’s neuroprotective mechanism in SCI through KEAP1/NRF2 pathway modulation. We demonstrate that MAG treatment robustly activates NRF2 signaling, enhancing downstream antioxidant gene expression. Using integrated in vivo and in vitro approaches, we show MAG administration: (i) Significantly reduces histopathological damage and improves functional recovery in SCI mice; (ii) Critically depends on NRF2 activation, as pharmacological NRF2 inhibition abrogates MAG’s benefits. These results establish MAG as a promising SCI therapeutic candidate and identify microglial mitochondrial rescue via NRF2 as a strategic approach to disrupt the neuroinflammation-oxidative stress cycle in neural trauma.

## 2. Materials and methods

### 2.1. Cell culture and treatment

BV-2 microglial cells, obtained from the Chinese Academy of Medical Sciences, were maintained in Dulbecco’s Modified Eagle Medium (DMEM; KeyGEN Biotech, Nanjing, China) containing 10% (v/v) fetal bovine serum (FBS; Gibco, Grand Island, NY, USA). Prior to assay, cells were conditioned with MAG (HY-76225, MedChemExpress, Weehawken, NJ, USA) at 1, 5, or 25 μg/mL for 24 h, followed by exposure to 1 μg/mL lipopolysaccharide (LPS; Sigma-Aldrich, St. Louis, MO, USA) for either 6 or 24 h to trigger an inflammatory phenotype. To assess the role of NRF2 in MAG’s effects, cells were pretreated with 5 μM ML385 (M408829, Aladdin Biochemical Technology, Shanghai, China), a selective NRF2 inhibitor, for 24 h prior to MAG exposure. All compounds were dissolved in dimethyl sulfoxide (DMSO; Beyotime), maintaining a final solvent concentration ≤0.1% (v/v) across conditions. Vehicle controls containing equivalent DMSO concentrations were included.

### 2.2. SCI Modeling and grouping

Eight-week-old male C57BL/6J mice (20–30 g) were procured from HuaChuang Sino (Taizhou, Jiangsu Province, China). All animal experiments were approved by the Animal Care Committee of Nantong University (Ethics Approval No.: S20250317-004) and conducted in accordance with the ARRIVE guidelines 2.0 and the National Institutes of Health (NIH) guidelines for ethical laboratory animal use. Mice were housed under specificpathogen-free conditions kept at constant temperature and humidity under a 12-h light/12-h dark cycle with food and water available ad libitum. Prior to the surgical procedure, each mouse was anesthetized via intraperitoneal injection of tribromoethanol (0.4 mg/g, M2940, Aibei Biotechnology, Nanjing, China). The dorsal skin at the T10 vertebral level was disinfected with povidone-iodine, and a standard laminectomy was performed[25]. After complete spinal cord exposure, a weight-drop impact device (RWD Life Science Co., Shenzhen, China) was used to induce contusion injury by dropping a 5 g weight from a height of 5 cm. Successful SCI model establishment was confirmed by observation of central hematoma formation in the spinal cord, accompanied by tail flick reflex and hindlimb paralysis. MAG was dissolved in a vehicle consisting of 10% (v/v) DMSO and 90% (v/v) saline containing 20% (w/v) SBE-β-CD to achieve concentrations of 50 and 100 mg/kg. Methylprednisolone (MP; HY-B0260, MedChemExpress, USA) was dissolved in the same vehicle at a concentration of 30 mg/kg[31]. Both drugs were administered intraperitoneally to SCI mice for 7 days following the injury, while an equivalent amount of vehicle was intraperitoneally injected into the remaining groups. The first administration of MAG was performed 6 h after SCI modeling. Manual urine expression was performed daily until spontaneous urination function was restored. The mice were randomly divided into the following five experimental groups: Group 1 (Vehicle control): T10 laminectomy with vehicle injection; Group 2 (SCI + Vehicle): Spinal cord injury (SCI) induction followed by vehicle treatment; Group 3 (SCI + 50 mg/kg MAG): SCI mice administered 50 mg/kg MAG; Group 4 (SCI + 100 mg/kg MAG): SCI mice administered 100 mg/kg MAG; Group 5 (SCI + 30 mg/kg MP): SCI mice administered 30 mg/kg MP.

### 2.3. Behavioral assessment

#### 2.3.1. Basso mouse scale (BMS) assessment

The motor function of the hindlimbs in mice with SCI was systematically evaluated using the BMS at 0, 1, 3, 7, 14, 21, and 28 days post injury (dpi). The assessment encompassed parameters such as ankle movement, coordination, paw position, trunk stability, and tail posture[32]. Scores range from 0 (complete flaccid paralysis) to 9 (full locomotor competence).

#### 2.3.2. Hindlimb reflex scoring method

Each mouse was gently lifted by its tail and held 30 cm above the bench for 14 s. Postural responses were rated on a 4-point scale[33]. 0: Normal hindlimb function (full extension); 1: Partial hindlimb extension impairment; 2: Complete hindlimb flexion (clasping reflex); 3: Total loss of hindlimb motor function. Each mouse was evaluated three times by at least two investigators blinded to treatment, and the mean of these six ratings was recorded as the final value.

#### 2.3.3. Footprint analysis

To evaluate forelimb and hindlimb motor function, mice were allowed to traverse a paper-lined runway on day 28 post-SCI. The forepaws and hindpaws were coated with non-toxic blue and red ink, respectively. Quantitative analysis focused on stride length and width, while qualitative observation was conducted to evaluate paw positioning, dragging patterns, and overall gait characteristics.

### 2.4. Histological staining

Following euthanasia via CO_2_ anesthesia, mice underwent cardiac perfusion with precooled PBS followed by 4% (w/v) paraformaldehyde. The collected organs – comprising the heart, hepatic tissue, spleen, pulmonary lobes, renal pair, brain, and spinal cord – were embedded in paraffin wax, and finally cut into serial sections of 5 µm thickness.

#### 2.4.1. Hematoxylin and eosin staining (H&E) staining

The procedure was conducted as per the manufacturer's protocol (G1076-500ML, Servicebio, Wuhan, China), involving staining with hematoxylineosin solution, followed by dehydration through a graded ethanol series and clearing in xylene. Histological analysis was performed using a Nikon microscope (Japan).

#### 2.4.2. Nissl staining

Nissl staining was performed using the Nissl Staining Kit (G1036-100ML, Servicebio, Wuhan, China). Following deparaffinization and graded rehydration, tissue slices were immersed for 5 min in a 0.5% (w/v) toluidine blue working solution to selectively reveal Nissl bodies. Differentiation was achieved using 0.1% (v/v) glacial acetic acid, followed by mounting with neutral balsam. A Nikon microscope was used to quantify surviving neurons in the spinal cord at 28 dpi.

#### 2.4.3. Luxol Fast Blue (LFB) staining

LFB staining was performed using the LFB Staining Kit (G1030-100ML, Servicebio, Wuhan, China). Tissue sections were incubated in LFB working solution for 40 min, followed by dehydration in absolute ethanol, and rinsing under running water. Nuclei were counterstained with hematoxylin, and myelin sheaths were specifically highlighted with LFB solution. After clearing in xylene for 5 min, sections were mounted with neutral balsam. Morphological assessment of myelin sheath integrity and pathological changes was conducted using a Nikon microscope.

### 2.5. RNA sequencing (RNA-Seq)

RNA-Seq reads first passed a quality audit via FastQC to confirm dataset reliability. Subsequently, adapter trimming was performed with Trimmomatic to remove sequencing adapters and low-quality bases. High-quality reads were then mapped to the appropriate mouse reference genome using STAR aligner with default parameters. Read-level gene quantification was carried out with featureCounts, while differential expression profiling was undertaken in DESeq2, adopting a stringent cut-off of Benjamini – Hochberg adjusted *p* < 0.05 together with |log₂ fold change| ≥ 1 to define significant transcripts. Functional interpretation was pursued with clusterProfiler, interrogating Gene Ontology (GO) terms and Kyoto Encyclopedia of Genes and Genomes (KEGG) annotations at an FDR threshold of 0.05. All inferential statistics were executed in R; group contrasts employed either the Student t-test or the Mann – Whitney U test, contingent on normality diagnostics, with α set to 0.05.

### 2.6. Molecular docking

Using AutoDock Vina, we performed in-silico docking to evaluate the binding affinity and interaction mode between the target protein and small-molecule compounds. Prior to docking, both the protein and ligands underwent extensive preparation, including the addition of hydrogen atoms, assignment of partial charges, and structural optimization. The entire protein was considered as the potential binding site, with the docking grid strategically centered at coordinates (x = 5.9 Å, y = −4.0 Å, z = −4.0 Å) within the protein structure. A cubic search space with an edge length of 100 Å was defined to ensure comprehensive sampling of potential binding conformations. The grid spacing was set to 0.375 Å to achieve a balance between computational efficiency and docking precision. Conformational sampling was performed using a genetic algorithm, followed by rigorous scoring based on binding affinity (kcal/mol). The generated poses were ranked according to their docking scores, and the top-scoring conformation was selected for subsequent binding mode analysis. Molecular visualization and interaction analysis were carried out using PyMOL (Version 3.0.3, Schrödinger LLC) and Discovery Studio 2019 (Dassault Systèmes).

### 2.7. Quantitative real-Time PCR (qPCR)

Total RNA was extracted from BV2 microglial cells using a TRIzol reagent (YFXM0011P; YIFEIXUE BioTech, Nanjing, China). Reverse transcription was conducted using the First Strand cDNA Synthesis Kit (YFXM0010; YIFEIXUE BioTech). The qPCR reaction system was prepared using template nucleic acids, specific primers (sequences detailed in Table 1), SYBR Green fluorescent dye, and other essential components. Each reaction mixture was carefully prepared to ensure consistency and accuracy. The samples were loaded into qPCR plates and amplified using a Bio-Rad Thermal Cycler (Hercules, CA, USA). Target mRNA abundance was normalized to β-actin, yielding internally consistent results. Data analysis was conducted with Bio-Rad CFX Manager software.

**Table 1.** The primers of qRT-PCR in the study.

Gene name	Forward sequence (5′−3′)	Reverse sequence (5′−3′)
IL-1β	GCAACTGTTCCTGAACTCAACT	ATCTTTTGGGGTCCGTCAACT
IL-6	TAGTCCTTCCTACCCCAATTTCC	TTGGTCCTTAGCCACTCCTTC
TNF-α	GACGTGGAACTGGCAGAAGAG	TTGGTGGTTTGTGAGTGTGAG
iNOS	GTTCTCAGCCCAACAATACAAGA	GTGGACGGGTCGATGTCAC
COX-2	TTCAACACACTCTATCACTGGC	AGAAGCGTTTGCGGTACTCAT

### 2.8. Western blotting (WB)

Mice spinal cords were rapidly dissected on ice, and total protein was solubilized using a commercial extraction reagent (KGB5303, KeyGEN Biotech); concentration was subsequently measured by a bicinchoninic-acid assay (KGB2101, KeyGEN Biotech). Equal protein loads were separated on SDS-polyacrylamide gels, electro-blotted onto polyvinylidene fluoride membranes (Millipore, USA), and non-specific sites were saturated with 5% (w/v) skim milk (BioFroxx, Guangzhou, China) to curtail background noise. Membranes were then exposed to primary antibodies (details provided in Table 2) at 4 °C overnight, rinsed in TBS-T, and incubated with horseradish-peroxidase-linked secondary antibodies for 60 min at room temperature. Bands were visualized with a TOUCH IMAGER XLi platform (E-Blot, Shanghai, China) and quantified in ImageJ (NIH, USA).

**Table 2.** Antibodies of interest in the study.

Antibodies name #Cat. No.	Source	Species	Application	Dilution rate
KEAP1 Polyclonal antibody #10503-2-AP	Proteintech	Rb	WB	1:1000
NRF2, NFE2L2 Polyclonal antibody #16396-1-AP	Proteintech	Rb	WB	1:1000
iNOS Polyclonal antibody #22226-1-AP	Proteintech	Rb	WB	1:1000
IL-1 beta Polyclonal antibody #26048-1-AP	Proteintech	Rb	WB	1:1000
IL-6 Rabbit pAb #500286	Zenbio	Rb	WB	1:1000
Beta Actin Monoclonal antibody #66009-1-Ig	Proteintech	Ms	WB	1:10000
Goat Anti-Mouse IgG (H + L), HRP #YFSA01	Yfxbio	Goat	WB	1:10000
Goat Anti-Rabbit IgG (H + L), HRP #YFSA02	Yfxbio	Goat	WB	1:10000
F4/80 Polyclonal antibody #28463-1-AP	Proteintech	Rb	IF	1:500
GFAP (GA5) Mouse mAb #3670	CST	Ms	IF	1:600
IL-1 beta Polyclonal antibody #26048-1-AP	Proteintech	Rb	IF	1:200
TNF-alpha Monoclonal antibody #60291-1-Ig	Proteintech	Ms	IF	1:200
IL6 Rabbit mAb #A21264 COX2/ Cyclooxygenase 2/ PTGS2 Monoclonal antibody #66351-1-Ig CoraLite488-conjugated Goat Anti-Rabbit IgG(H + L) #SA00013-2 CoraLite594 – conjugated Goat Anti-Mouse IgG(H + L) #SA00013-3 Donkey anti-Rabbit IgG (H + L) Highly Cross-Adsorbed Secondary Antibody, Alexa Fluor™ 568 #A10042 Donkey anti-Rabbit IgG (H + L) Highly Cross-Adsorbed Secondary Antibody, Alexa Fluor™ 568	Abcam Proteintech Proteintech Proteintech Thermo Fisher Thermo Fisher	Rb Ms Goat Goat Goat Goat	IF IF IF IF IF IF	1:200 1:200 1:500 1:500 1:500 1:500

### 2.9. Immunofluorescence (IF) staining

Sections underwent deparaffinization in xylene and rehydration through an ethanol gradient. Heat-induced epitope retrieval was performed in citrate buffer, followed by 3% (v/v) H₂O₂ treatment to inactivate endogenous peroxidase. After blocking nonspecific sites with 5% (w/v) bovine serum albumin(BSA, Beyotime) for 60 min, the sections were sequentially incubated with primary antibodies (details in Table 2) and their corresponding fluorophore-conjugated secondary antibodies. Nuclei were counterstained with 4′,6-diamidino-2-phenylindole (DAPI, Beyotime) before the slides were mounted with an antifade medium. Cultured cells were fixed with 4% (w/v) paraformaldehyde (15 min), permeabilized with 0.1% (v/v) Triton X-100 (5 min), blocked with 5% (w/v) BSA (30 min), and subjected to the same immunofluorescence protocol as tissue sections. Fluorescence images were acquired using either immunofluorescence microscopy or confocal laser scanning microscopy. High-resolution imaging systems were employed to visualize the colocalization of different indicators.

### 2.10. Mitochondrial membrane potential (ΔΨm) measurement

Mitochondrial membrane potential (ΔΨm) in BV-2 microglia was evaluated with a JC-1 probe (M34152, Thermo Fisher Scientific, Waltham, MA, USA). After seeding on confocal dishes, cells were incubated with the JC-1 working solution at 37 °C for 20 min under dark conditions. Carbonyl cyanide m-chlorophenylhydrazone (CCCP) was applied to parallel wells to establish a positive control for depolarization. Fluorescence imaging was performed using confocal microscopy. The red-to-green fluorescence intensity ratio serves as an indicator of mitochondrial functional status.

### 2.11. Measurement of mitochondrial reactive oxygen species (ROS)

Mitochondrial superoxide generation in BV-2 microglia was monitored with the MitoSOX™ Red probe (M36007, Thermo Fisher Scientific, Waltham, MA, USA). After seeding on confocal dishes, cells were loaded with the dye at 37 °C for 30 min in the dark. Parallel wells treated with CCCP were established as a positive control for mitochondrial ROS production. After two washes with PBS, live-cell imaging was performed using a confocal microscope. The mean fluorescence intensity of MitoSOX™ Red was quantitatively analyzed to assess mitochondrial ROS production.

### 2.12. Statistical analysis

All quantitative results are presented as mean ± standard error of the mean (SEM) and were visualized with GraphPad Prism 10.0 (Version 10.0, GraphPad Software, San Diego, CA, USA). A *p*-value < 0.05 was considered statistically significant. The normality of data distribution was assessed with the Shapiro–Wilk test, and the homogeneity of variances was verified using the Brown-Forsythe test. For comparisons between two independent groups that met the assumptions of normality and equal variance, an unpaired two-tailed Student's t-test was used; otherwise, the Mann–Whitney U test was applied. For multi-group comparisons, a two-way analysis of variance (ANOVA) followed by Tukey's post hoc test was employed for parametric data, while the Kruskal–Wallis test with Dunn's post hoc correction was used for non-parametric data.

## 3. Results

### 3.1. MAG alleviates pathological damage and accelerates motor recovery after SCI

**Figure 1.**
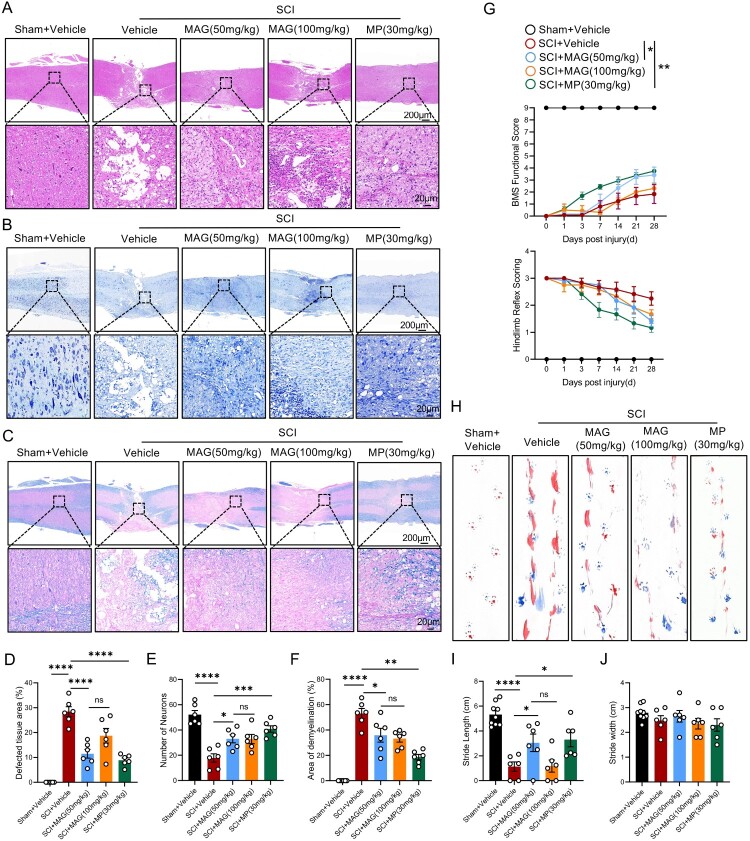
(A-C) Histopathological staining (HE, Nissl, LFB) of spinal cord tissues at 28 dpi; scale bar = 200/20 μm. (D-F) Quantitative analysis of lesioned tissue areas, neuronal counts and demyelinated regions (n = 6). (G) Functional assessments: BMS scores and hind-limb reflex tests at 0, 1, 3, 7, 14, 21, and 28 dpi (n ≥ 6). (H) Footprint images at 28 dpi. (I-J) Quantitative analysis of stride length and width from gait analysis (n ≥ 6). Data are expressed as the mean ± SEM. (*p < 0.05; **p < 0.01; ***p < 0.001; ****p < 0.0001; ns = No significance).

Before investigating the role of MAG in promoting neurological recovery after SCI, we first validated that systemic administration of MAG is acceptable in terms of safety (Suppl. Figure 1). To evaluate the neuroprotective effects of MAG following SCI, histological assessments were conducted at 28 dpi using HE, Nissl, and LFB staining (Figure 1A-C). Interestingly, HE staining revealed that MAG treatment significantly reduced the lesion area in spinal cord tissue, with the 50 mg/kg MAG group showing more pronounced tissue repair. Moreover, Nissl staining demonstrated increased neuronal survival around the injury site, while LFB staining indicated decreased demyelination in the 50 mg/kg MAG-treated group (Figure 1D-F). Notably, the therapeutic efficacy of 50 mg/kg MAG was comparable to that of 30 mg/kg MP, which was included as a positive control (Figure 1D-F). The BMS results showed no significant differences between groups at 14 dpi. However, at 21 and 28 dpi, mice treated with 50 mg/kg MAG exhibited significantly higher BMS scores compared to untreated controls. The improved hindlimb reflex scores in the 50 mg/kg MAG group at 28 dpi further confirmed the positive effects of MAG on functional recovery after SCI (Figure 1G). Gait analysis revealed that SCI mice treated with 50 mg/kg MAG exhibited significantly increased stride length and reduced hindlimb dragging compared to the injury group, although no significant differences in stride width were observed among groups (Figure 1H-J). Similarly, 30 mg/kg MP treatment led to notable functional improvements, paralleling the effects observed with 50 mg/kg MAG (Figure 1G-J). These results conclusively demonstrate that 50 mg/kg MAG effectively accelerates motor recovery after spinal cord trauma.

### 3.2. MAG alleviates microglia-induced neuroinflammation and oxidative stress after SCI

**Figure 2.**
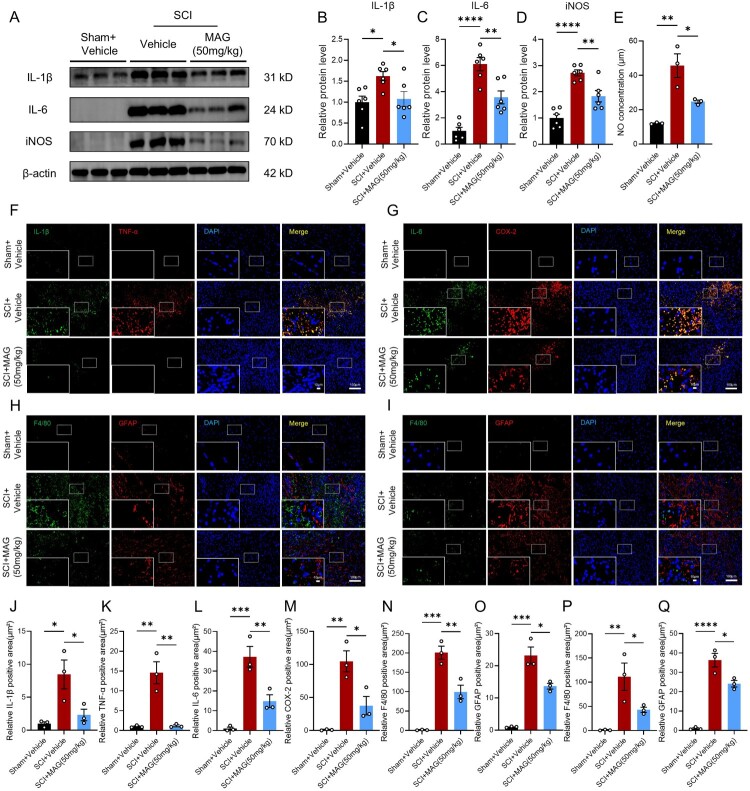
(A) Western blotting analysis of IL-1β (31 kD), IL-6 (24 kD), and the monomeric form of iNOS (70 kD) in SCI mice at 3 dpi, with β-actin (42kD) as loading control. (B-D) Quantitative analysis of IL-1β, IL-6, iNOS (monomer) protein levels (n = 6). (E) NO production (n = 3). (F) IF co-staining of IL-1β (green) and TNF-α (red); scale bar = 10/100 μm. (G) IF co-staining of IL-6 (green) and COX-2 (red); scale bar = 10/100 μm. (H-I) IF co-staining of F4/80 (green) and GFAP (red) at 7/28 dpi; scale bar = 10/100 μm. (J-M) Quantitative analysis of the relative positive area of IL-1β, TNF-α, IL-6 and COX-2 (n = 3). (N-O) Quantitative analysis of the relative positive area of F4/80 and GFAP at 7 dpi (n = 3). (P-Q) Quantitative analysis of the relative positive area of F4/80 and GFAP at 28 dpi (n = 3). Data are expressed as the mean ± SEM. (*p < 0.05; **p < 0.01; ***p < 0.001; ****p < 0.0001).

During SCI pathogenesis, activated microglia excessively release iNOS, COX-2, and pro-inflammatory cytokines, amplifying neuroinflammatory responses and inducing oxidative stress. Western blot analysis demonstrated significant upregulation of iNOS and pro-inflammatory cytokines like IL-1β and IL-6 at 3 dpi. MAG treatment significantly mitigated SCI-induced upregulation of iNOS, IL-1β, and IL-6, highlighting its potent inhibitory effects on neuroinflammation following SCI (Figure 2A-D, Suppl. Figure 2). To functionally correlate iNOS expression with its enzymatic activity, we measured nitric oxide (NO) levels, which are primarily catalyzed by the active dimeric form of iNOS. Consistent with the protein expression data, a significant increase in NO metabolites was observed in the SCI group, and this increase was markedly attenuated by MAG treatment (Figure 2E). IF staining further corroborated MAG's suppressive effects on microglia-mediated neuroinflammation and oxidative stress. Quantitative IF analysis revealed that 50 mg/kg MAG treatment significantly reduced the fluorescence intensity of IL-1β, TNF-α, IL-6, and COX-2 in SCI-injured tissue compared to vehicle-treated controls (Figure 2F-G, 2J-M). The progression of secondary neuroinflammatory damage following SCI was evidenced by pronounced glial scar formation, which ultimately impaired functional recovery. Immunofluorescence quantification demonstrated that 50 mg/kg MAG treatment at both 7 and 28 dpi markedly diminished the F4/80-positive microglial territory and the GFAP-positive astrocytic area relative to the SCI group (Figure 2H-I, 2N-Q). These findings suggest that MAG modulates post-SCI neuroinflammation and glial scar formation by regulating microglial and astrocytic activation, thereby fostering a neuroprotective microenvironment conducive to neural repair. Collectively, these results demonstrate that MAG treatment not only mitigates aberrant microglial activation but also effectively attenuates inflammation-mediated secondary damage through coordinated modulation of glial reactivity.

### 3.3. MAG suppresses LPS-stimulated neuroinflammation and oxidative stress in BV-2 microglia in vitro

**Figure 3.**
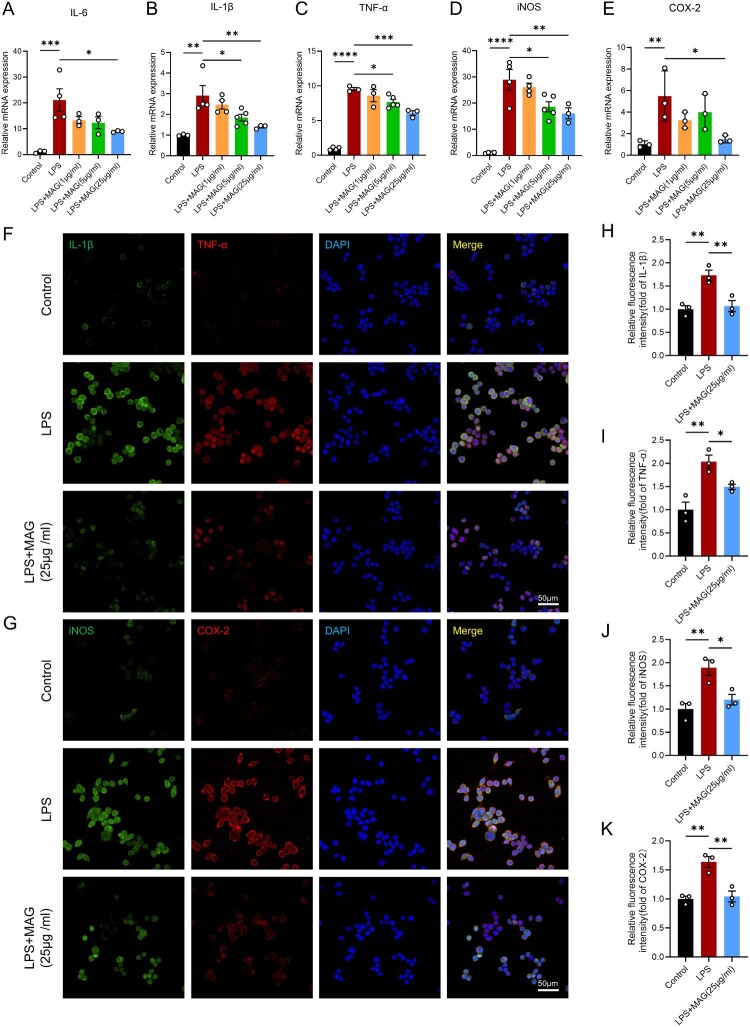
(A-E) Relative mRNA levels of IL-1β, IL-6, TNF-α, iNOS and COX-2 in BV-2 microglia stimulated with LPS after 24h-treatment with MAG. (F) IF co-staining of IL-1β (green) and TNF-α (red); scale bar = 50 μm. (G) IF co-staining of iNOS (green) and COX-2 (red); scale bar = 50 μm. (H-K) Quantitative analysis of the relative positive area of IL-1β, TNF-α, IL-6 and COX-2 (n = 3). Data are expressed as the mean ± SEM (*p < 0.05; **p < 0.01; ***p < 0.001; ****p < 0.0001).

To mimic neuroinflammation after traumatic spinal cord injury in vitro, BV-2 microglia were challenged with LPS, thereby reproducing the cytokine milieu observed post-SCI. PCR analysis demonstrated that LPS significantly increased mRNA levels of IL-1β, IL-6, TNF-α, iNOS, and COX-2, successfully establishing a microglial neuroinflammation and oxidative stress model. Exposure to MAG at 25 µg/mL substantially attenuated the LPS-driven up-regulation of these genes (Figure 3A-E), whereas neither 1 μg/ml nor 5 μg/ml MAG showed significant effects on their expression. IF staining further confirmed that 25 μg/ml MAG significantly attenuated LPS-induced expression of IL-1β, TNF-α, iNOS, and COX-2 (Figure 3F-K). These results collectively demonstrate the potent inhibitory effects of MAG on microglia-mediated neuroinflammation and oxidative stress in vitro. Therefore, in subsequent cell experiments, we proceeded with 25 μg/ml MAG for further investigation.

### 3.4. MAG regulates expression of mitochondrial and oxidative stress gene clusters during microglial neuroinflammation

**Figure 4.**
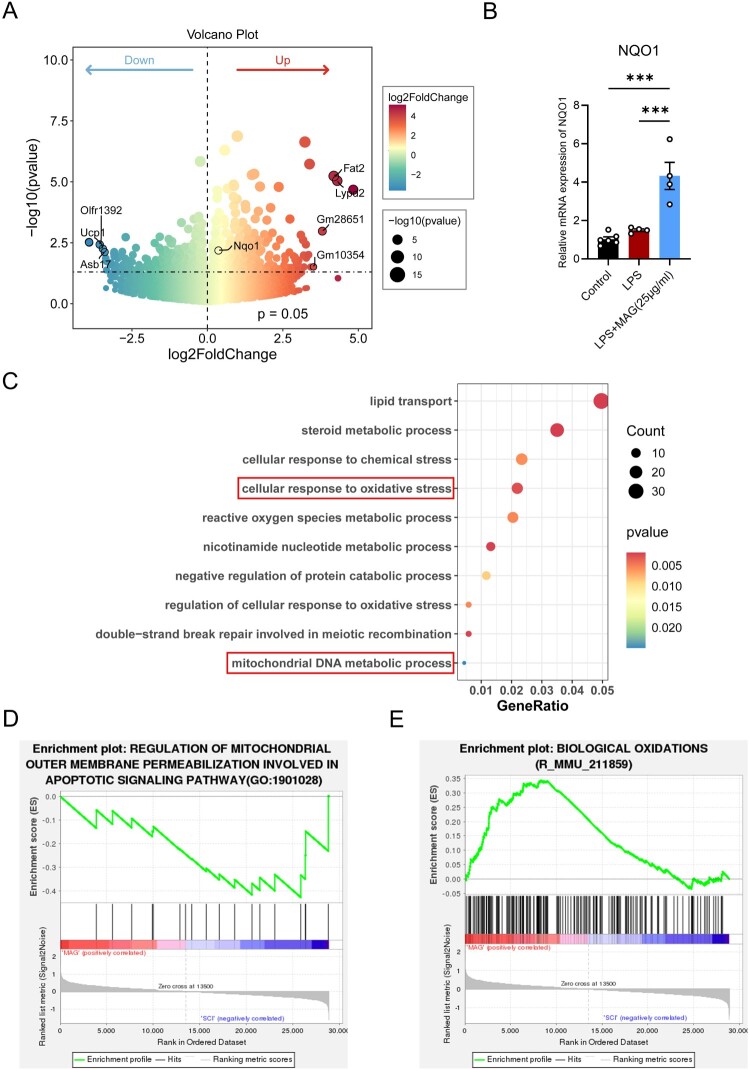
(A) Volcano plot of DEGs between the SCI + MAG (50 mg/kg) group and the SCI group, highlighting upregulated antioxidant gene NQO1. (B) Relative mRNA level of NQO1 in LPS-challenged BV-2 microglia exposed to MAG for 24 h. (C) GO enrichment analysis of DEGs, focusing on biological processes. (D-E) Enrichment plots. Data are expressed as the mean ± SEM. (***p < 0.001).

RNA-Seq analysis revealed that 50 mg/kg MAG treatment induced significant transcriptional changes. Volcano plot analysis identified 438 upregulated genes and 443 downregulated genes, among which NAD(P)H: quinone oxidoreductase 1 (NQO1) was one of the notable differentially expressed genes, showing a significant upward trend after MAG treatment (Figure 4A). Positioned downstream of KEAP1 – NRF2 signaling, the enzyme NQO1 operates as a pivotal antioxidant that neutralizes cytosolic ROS and sustains redox equilibrium[34–37]. Quantitative PCR revealed that exposure of LPS-activated BV2 cells to 25 µg/ml MAG significantly elevated NQO1 transcript abundance (Figure 4B). GO enrichment further demonstrated that these differentially expressed genes clustered within processes such as ‘response to oxidative stress’ and ‘mitochondrial DNA metabolism’ (Figure 4C), implying that MAG modulates oxidative balance and mitochondrial integrity. Additionally, enrichment plots further confirmed the significant enrichment of pathways such as ‘regulation of mitochondrial apoptotic signaling pathway’ and ‘biological oxidations’ (Figure 4D-E). Collectively, these data support the hypothesis that MAG confers anti-inflammatory and antioxidant effects by regulating the KEAP1/NRF2/NQO1 pathway.

### 3.5. MAG alleviates mitochondrial dysfunction by modulating the KEAP1/NRF2/NQO1 pathway

**Figure 5.**
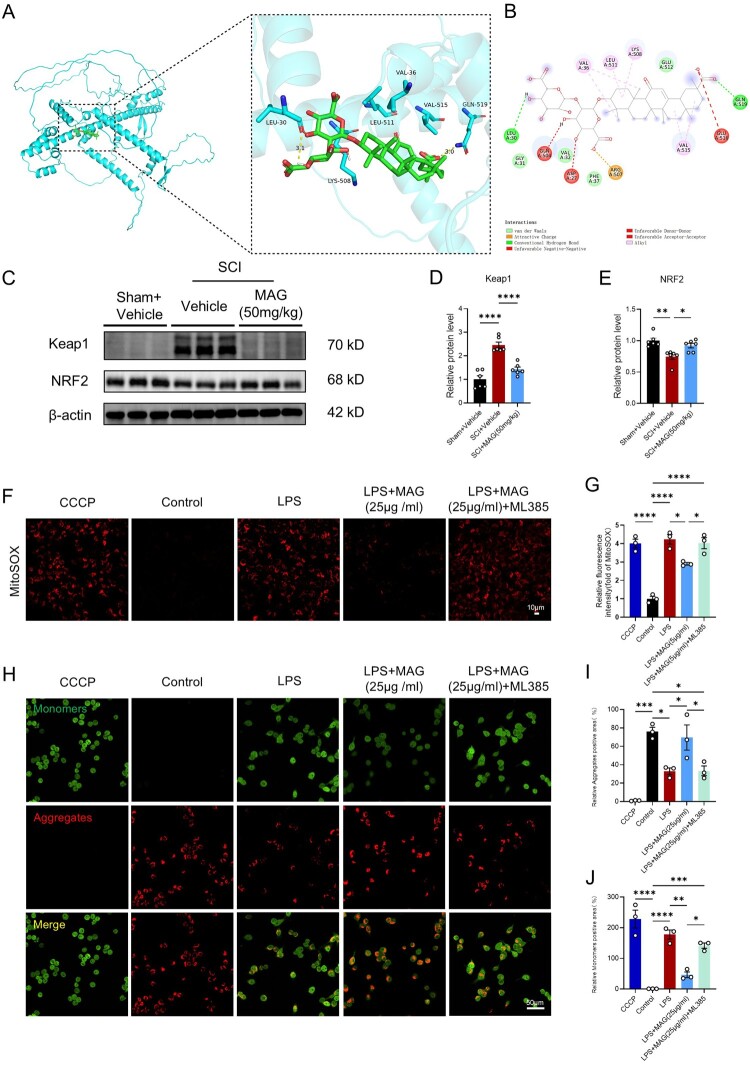
(A-B) Molecular docking revealed the binding mode of NRF2 and MAG within the 3D structure and the electrostatic surface characteristics of the protein. (C) Western blot of Keap1 (70kD) and NRF2 (68kD) in SCI mice at 3 dpi. (D-E) Quantitative analysis of Keap1 and NRF2 protein levels (n = 6). (F) Mitochondrial ROS were visualized with red MitoSOX (red) (n = 3); scale bar = 10 μm. (G) Quantitative analysis of ROS levels. (H) Microglial ΔΨm was assessed by JC-1 (n = 3); scale bar = 50 μm. (I-J) Quantitative analysis of aggregates(red)/ monomers(green) levels. Data are expressed as the mean ± SEM. (*p < 0.05; **p < 0.01; ***p < 0.001; ****p < 0.0001).

Based on the RNA-Seq results, we analyzed the interaction between MAG and NRF2 through molecular docking. The results demonstrated a high-affinity binding (binding energy of −8.6 kcal/mol), primarily through the formation of hydrogen bonds with Leu30 (3.1 Å) and Gln519 (3.0 Å), as well as hydrophobic interactions with residues such as Lys508 and Leu511 (Figure 5A-B). This stable intermolecular interaction provides a structural foundation for subsequent functional studies. Validation via Western blot analysis showed that KEAP1 protein expression was significantly upregulated, while NRF2 expression was downregulated. Importantly, these abnormalities were significantly reversed by MAG treatment (Figure 5C-E).

Next, we used LPS stimulation to induce oxidative stress in BV2 microglia. Detection using the MitoSOX probe revealed a marked increase in mitochondrial ROS level, which were effectively reduced by MAG treatment. To investigate the role of NRF2 in this process, we introduced the specific NRF2 inhibitor ML385 for intervention. The results showed that ML385 treatment significantly reversed the inhibitory effect of MAG on LPS-induced elevation of mitochondrial ROS(Figure 5F-G), causing the mitochondrial ROS level to rise to a level comparable to that of the LPS group. This finding suggests that the inhibitory effect of MAG on mitochondria-induced oxidative stress is partially dependent on the NRF2 signaling pathway. Further assessment of ΔΨm using the JC-1 probe revealed that LPS treatment led to significant depolarization of ΔΨm. Notably, MAG intervention significantly ameliorated this damage, indicating that MAG exerts a protective effect on mitochondrial function in microglia under inflammatory conditions. However, ML385 pretreatment weakened the protective effect of MAG on ΔΨm, exacerbating the degree of ΔΨm depolarization (Figure 5H-J). Taken together, the data highlight the KEAP1/NRF2/NQO1 axis as a pivotal mediator through which MAG safeguards mitochondrial integrity and curbs neuroinflammation.

### 3.6. MAG suppresses mitochondrial dysfunction-induced inflammation in microglial cells via a NRF2-dependent mechanism

**Figure 6.**
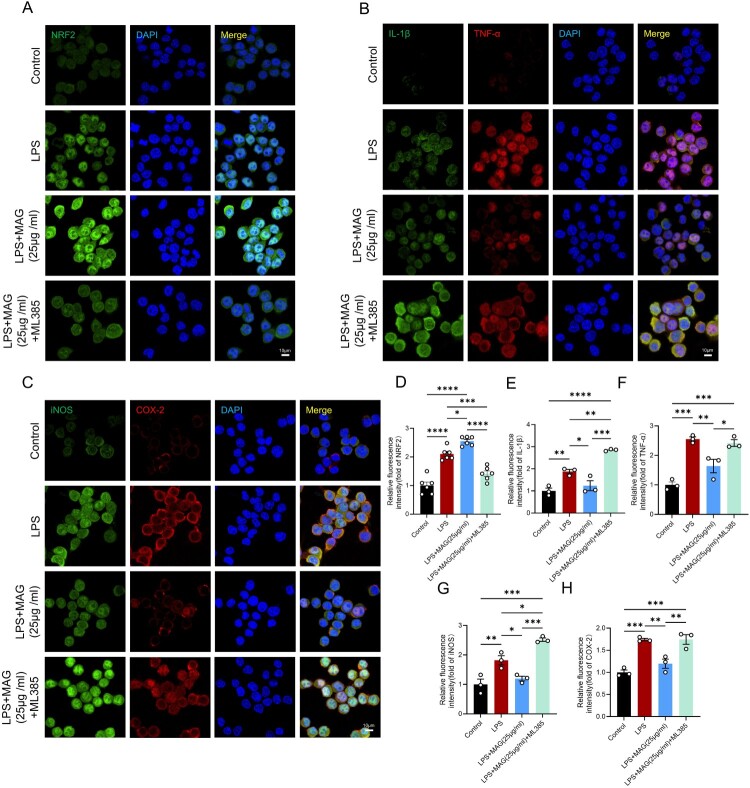
(A) IF staining of NRF2 (green); scale bar = 10 μm. (B) IF co-staining of IL-1β (green) and TNF-α (red); scale bar = 10 μm. (C) IF co-staining of iNOS (green) and COX-2 (red); scale bar = 10 μm. (D) Quantitative analysis of the relative fluorescence intensity of NRF2 (n = 6). (E-H) Quantitative analysis of the relative fluorescence intensity of IL-1β, TNF-α, IL-6 and COX-2 (n = 3). Data are expressed as the mean ± SEM. (*p < 0.05; **p < 0.01; ***p < 0.001; ****p < 0.0001).

To further elucidate the molecular mechanism underlying MAG’s inhibition of microglial activation via NRF2, we continued to perform functional validation using ML385, a specific NRF2 inhibitor. Immunofluorescence analysis demonstrated that treatment with 5 μM ML385 significantly attenuated the MAG-induced nuclear translocation of NRF2 (Figure 6A, 6D). Functional rescue assays corroborated our earlier observations: MAG potently dampened LPS-evoked up-regulation of IL-1β, TNF-α, iNOS and COX-2. Yet, concurrent administration of the NRF2 inhibitor ML385 largely overturned this suppression, causing a pronounced rebound in inflammatory transcripts (Figure 6B-C, 6E-H). Collectively, the pharmacological blockade data establish that MAG’s neuroprotection is mediated through NRF2 induction and fine-tuning of the KEAP1/NRF2/NQO1 axis, thereby attenuating neuroinflammation, limiting oxidative damage, and preserving mitochondrial integrity.

## 4. Discussion

SCI is often complicated by secondary pathological processes, marked by progressively exacerbated neuroinflammatory responses, oxidative stress damage, and mitochondrial dysfunction. These pathological features have been clinically confirmed as significant contributors to poor patient outcomes[38]. Methylprednisolone (MP), currently the most widely used anti-inflammatory agent in clinical practice, mitigates secondary injury by suppressing inflammatory responses and lipid peroxidation during the acute phase[39, 40]. However, its high-dose (30 mg/kg) and short-term (≤8 h) administration is associated with numerous adverse effects, including hyperglycemia, increased risk of infection, gastrointestinal bleeding, and immunosuppression[41]. Despite the administration of high-dose pulse regimens, methylprednisolone (MP) fails to achieve therapeutically relevant concentrations at the lesion site because intact areas of the blood – spinal cord barrier(BSCB) impede its penetration, markedly constraining its clinical effectiveness [42, 43].

MAG, an extract derived from glycyrrhiza uralensis root, has been reported to improve liver function, mitigate inflammatory responses, inhibit oxidative stress, and regulate gut microbiota[44–47]. Our selection of MAG doses (50 and 100 mg/kg) was based on prior studies of glycyrrhizic acid derivatives in inflammatory models, and systemic safety assessment revealed no overt toxicity at these doses (Suppl. Figure 1). Notably, the 50 mg/kg dose exhibited superior therapeutic efficacy compared to 100 mg/kg, suggesting a potential bell-shaped dose–response curve, which is not uncommon for natural compounds. Further dose-ranging and pharmacokinetic studies are warranted to optimize dosing regimens. Despite extensive research on the pharmacological effects of MAG, studies focusing on its anti-inflammatory and antioxidant properties in SCI remain limited. Our findings demonstrate that MAG effectively suppresses the production of inflammatory mediators (iNOS, COX-2), reduces the generation of pro-inflammatory cytokines (IL-1β, IL-6, TNF-α), and significantly decreases ROS generation both in vitro and in vivo. These results suggest that MAG can inhibit microglia-driven neuroinflammation and oxidative stress following SCI. Furthermore, as a small-molecule compound, MAG readily crosses the BSCB upon intraperitoneal injection, addressing the issue of insufficient local anti-inflammatory drug delivery. Beyond suppressing the expression of inflammatory mediators, our study provides deeper insight into the effect of MAG on iNOS function. Western blot analysis revealed that SCI induced the expression of both the monomeric (70 kD) and, importantly, the dimeric (130–140 kD) form of iNOS. The formation of the homodimer is essential for iNOS enzymatic activity and NO production. MAG treatment effectively suppressed the levels of both forms. Critically, this reduction in the active dimer was functionally corroborated by a significant decrease in NO metabolite levels in MAG-treated SCI mice. This compelling correlation between the reduction of the active iNOS dimer and the decrease in NO output solidifies its functional impact on a key inflammatory pathway.

The KEAP1/NRF2 pathway is a central regulatory hub for cellular oxidative stress responses. Its activation can induce the expression of the antioxidant enzyme NQO1, thereby restoring cellular redox homeostasis[48, 49]. Through RNA sequencing and molecular docking techniques, this study found that MAG directly binds to the NRF2 protein (with a binding energy of −8.6 kcal/mol), blocks KEAP1-mediated ubiquitination and degradation, promotes the nuclear translocation of NRF2, and initiates the downstream NQO1 antioxidant program. Notably, MAG not only inhibits the release of inflammatory cytokines but also enhances endogenous antioxidant capacity, fundamentally blocking the pathological cascade of ‘oxidative stress – mitochondrial damage.’ It further reduces mitochondrial ROS released by overactivated microglia, restores ΔΨm, and protects mitochondrial function to the greatest extent possible. Moreover, by activating the NRF2 pathway, MAG elicits a concerted action that addresses both inflammatory and oxidative stress components of secondary injury. This mechanism-based approach, rooted in enhancing cellular defense systems, may offer a more comprehensive therapeutic advantage over the symptomatic suppression achieved by MP.

Numerous studies have confirmed that microglia-mediated neuroinflammation and oxidative stress following SCI promote the release of neurotoxic substances[50–52]. This pathological process not only exacerbates excessive glial scar formation but also significantly inhibits axonal regeneration, further aggravating secondary damage to neural tissue[53–55]. Such cascading effects directly impair motor function recovery. To systematically evaluate the therapeutic efficacy of MAG, this study employed a 7-day intraperitoneal injection regimen and assessed neural tissue repair and motor function recovery in SCI mice using multidimensional metrics. Histological analysis revealed significant improvements in the MAG-treated group compared to the injury-only group, including reduced neural tissue defect area, increased neuronal survival rate, and markedly alleviated demyelination. In terms of functional recovery, MAG-treated mice exhibited significantly improved hindlimb motor function scores, enhanced gait coordination, and shortened reflex recovery time. Importantly, the therapeutic efficacy of 50 mg/kg MAG was comparable to that of 30 mg/kg MP, yet without the associated adverse effects, highlighting MAG’s superior safety and tolerability profile. These results not only confirm the remarkable efficacy of MAG in promoting neural tissue repair but also highlight its potential clinical value in improving functional prognosis.

Although the present work establishes MAG’s neuroprotective efficacy in spinal cord injury, pivotal issues remain that demand deeper exploration. First, although our findings revealed a correlation between MAG administration and NRF2 activation, the precise molecular mechanisms governing this interaction remain to be elucidated. Future studies should employ additional methodologies beyond pharmacological inhibition (e.g. ML385) to fully characterize this relationship. Second, the long-term therapeutic efficacy and safety profile of MAG in the context of SCI require rigorous evaluation, particularly with respect to potential toxicological effects. Although the dose selection in this study was informed by prior proof-of-principle studies with glycyrrhizic acid derivatives, future work must include a comprehensive pharmacokinetic and metabolism analysis to optimize dosing and enable clinical translation. Ultimately, systematic investigation is required to determine whether MAG engages additional pathophysiological axes – mitochondrial impairment being a prominent example – and whether its efficacy can be potentiated through co-administration with complementary therapeutics or advanced biomaterials. Answering these outstanding questions will yield deeper insight into the therapeutic promise of MAG and clarify its translational relevance for clinical practice.

Our study provides the first experimental evidence that MAG attenuates microglia-mediated neuroinflammation, thereby promoting neural tissue preservation and enhancing motor function recovery in a murine model of SCI. Mechanistically, MAG exerts its therapeutic effects through multiple mechanisms, including suppression of microglia-induced neuroinflammation and oxidative stress, stabilization of ΔΨm, preservation of mitochondrial bioenergetic function, and upregulation of the KEAP1/NRF2 signaling pathway after SCI. The present data reinforce MAG as a promising candidate for spinal cord injury therapy and, more broadly, illuminate anti-inflammatory avenues that may accelerate neural repair and regeneration.

## Supplementary Material

Supplementary (1).docx

## Data Availability

The datasets generated and analyzed during this study are available from the corresponding author upon reasonable request.
