# Supplementary material for: Monoammonium glycyrrhizinate ameliorates mitochondrial dysfunction-mediated oxidative stress and neuroinflammation via the NRF2/NQO1 axis after spinal cord injury
Source: Redox Rep. 2025 Nov 15;30(1):2585221. doi: 10.1080/13510002.2025.2585221 (PMC12621352; doi:10.1080/13510002.2025.2585221)
Supplement: Supplementary (1).docx [file YRER_A_2585221_SM8287.docx]

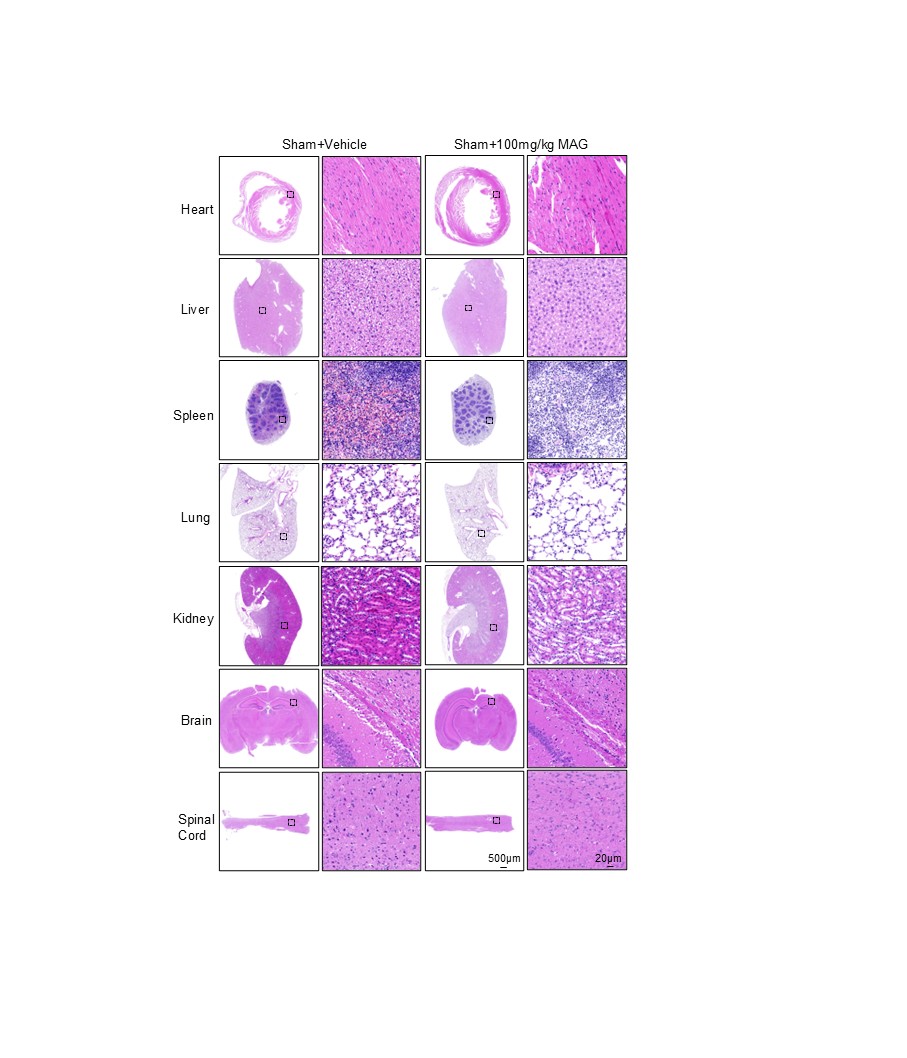


**Suppl. Figure 1** HE staining of heart, liver, spleen, lung, kidney, brain, and spinal cord tissues from Sham-operated mice treated with 100mg/kg MAG or not. Scale bar = 500/20 μm.


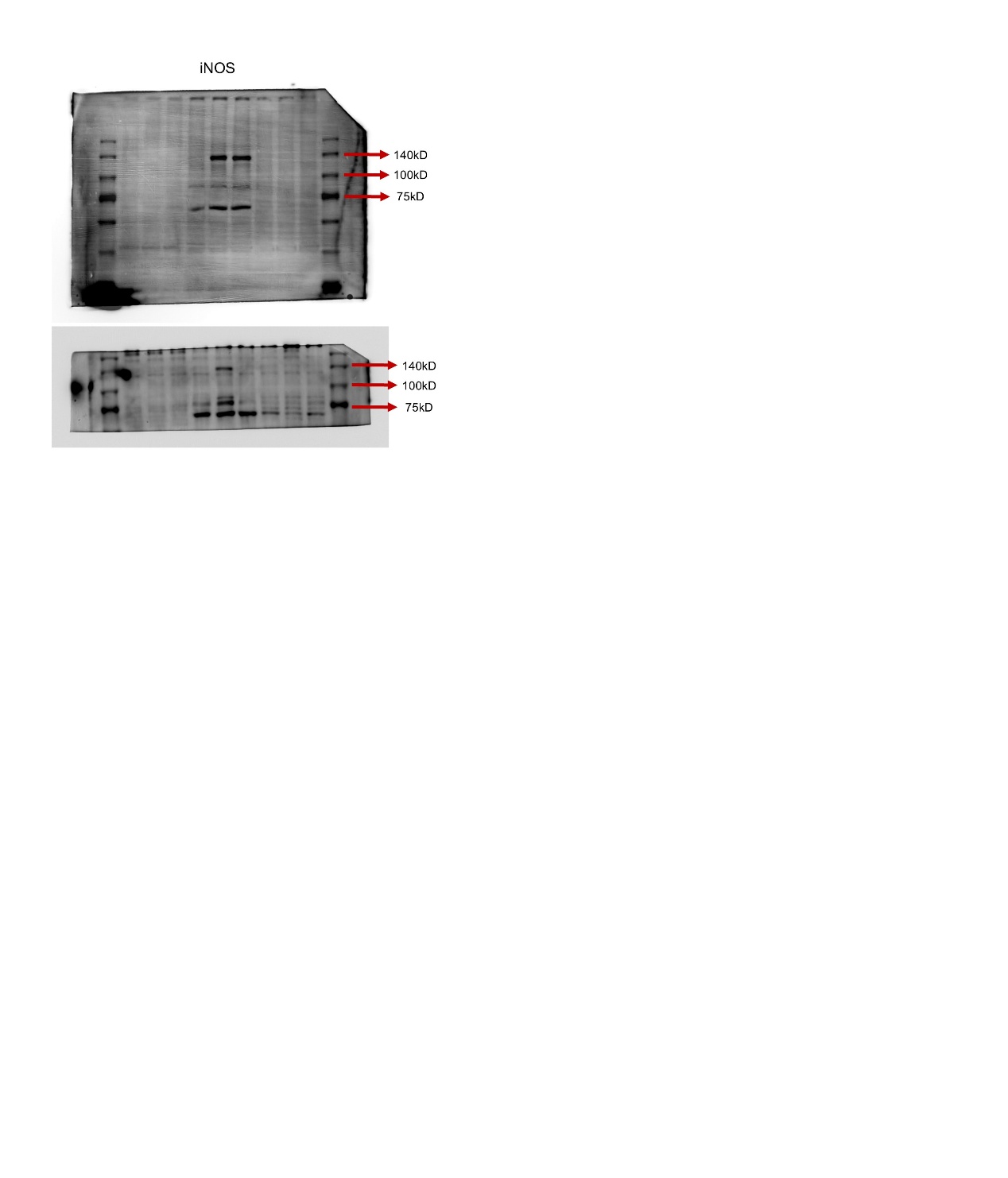


**Suppl. Figure 2** Western blot analysis of iNOS expression in SCI mice at 3 dpi. The blot shows the full membrane, detecting both the iNOS dimer at 130-140 kD and the monomer at 70 kD.
